# How ‘Idiopathic’ Is Adolescent Idiopathic Scoliosis? A Systematic Review on Associated Abnormalities

**DOI:** 10.1371/journal.pone.0097461

**Published:** 2014-05-12

**Authors:** Tom P. C. Schlösser, Geert J. M. G. van der Heijden, Anne L. Versteeg, René M. Castelein

**Affiliations:** 1 Department of Orthopaedic Surgery, University Medical Center Utrecht, Utrecht, the Netherlands; 2 Department of Epidemiology, University Medical Center Utrecht, Utrecht, the Netherlands; 3 Department of Social Dentistry, Academic Center for Dentistry Amsterdam, VU Amsterdam University and University of Amsterdam, Amsterdam, the Netherlands; Van Andel Institute, United States of America

## Abstract

**Background:**

Despite more than a century of dedicated research, the etiology and pathogenesis of adolescent idiopathic scoliosis (AIS) remain unclear. By definition, ‘idiopathic’ implies an unknown cause. Nevertheless, many abnormalities concomitant to AIS have been described, often with the suggestion that these abnormalities are related to etio-pathogenesis. Insight in the concomitant abnormalities may assist in improving the understanding of the etiological pathways of AIS. We aimed to systematically review and synthesize available studies on abnormalities concomitant to AIS.

**Methods:**

Original studies comparing untreated AIS patients with healthy adolescents on abnormalities other than the deformity of the spine were retrieved from PubMed and Embase. We followed PRISMA guidelines and to quantify the relationship between each abnormality and AIS we used a best-evidence-syntheses for relating risk-of-bias to consistency of effect sizes.

**Results:**

We identified 88 relevant citations, forty-seven carried high risk-of-bias and twenty studies did not report quantitative data in a sufficient manner. The remaining twenty-one publications failed to report data from before initiation of the deformity and blind assessments. These cross-sectional studies provided data on fourteen abnormalities concomitant to AIS. With our best-evidence-syntheses we were unable to find both strong evidence and a consistent pattern of occurrence for AIS and any of these abnormalities. From moderate risk-of-bias studies a relatively consistent pattern of occurrence for AIS and impaired gait control (4 studies; 155 subjects; Cohen’s *d* = 1.00) and decreased bone mineral density (2 studies; 954 subjects; Cohen’s *d* = −0.83) was found. For nine abnormalities a consistent pattern of occurrence with AIS was found, but the evidence for these was weak.

**Conclusions:**

Based on the available literature, strong evidence is lacking for a consistent pattern of occurrence of AIS and any abnormality. The relevance for understanding the multifactorial etiology of AIS is very limited.

## Introduction

Scoliosis is a complex three-dimensional deformity of the spine.[1] Although there are many forms of scoliosis, most cases (84–89%) develop without a known cause, in previously healthy girls during the adolescent growth spurt: this is called adolescent idiopathic scoliosis (AIS).[2], [3] 0.5–3% of the population is affected by AIS, no early preventive treatment is available and more than 10% of the patients require intensive brace therapy or invasive surgical correction.[3]–[5] While these challenges call for better understanding of the natural history of AIS, the etiology of AIS remains enigmatic.

Although the term ‘idiopathic’ (<$>\raster(70%)="rg1"<$>δι*ο*ς = one’s own and πάθ*ο*ς = suffering) implies that the cause of AIS is unknown and that the patient is otherwise -except for the spinal deformity- normal, many subtle abnormalities have been reported in the literature. It has been suggested that these findings could be helpful in clarifying the etiology of the disorder.[6] Even limited evidence for an abnormality in AIS patients has sometimes led to postulation of a new hypothesis on the role of the studied abnormality as unique causal factor of AIS.[7], [8] Clinicians treating patients with AIS, however, are often impressed with the absolutely normal development of the patient, both intellectually and physically, up to the moment that the spine starts to grow crooked and it is hard to understand the relevance of many of the reported more general and systemic abnormalities. In addition, evidence exists that intrinsic spinal biomechanics of the upright human spine, as well as genetics play a key role in the multifactorial causation of different pediatric spinal deformities, such as idiopathic scoliosis [9]–[12].

Insight in the concomitant abnormalities may assist in determining their relevance and to improve understanding of the etiological pathways of AIS. The different etiological hypotheses and genetic abnormalities have already been discussed in systematic reviews by Kouwenhoven et al., Lowe et al. and a recent review of Gorman et al., respectively.[7]–[9] Nevertheless, the epidemiology and relevance of the enormous number of phenotypical abnormalities in AIS patients have not been assessed to date. The purpose of this review, therefore, is to give as complete an overview as possible of all abnormalities, other than the spinal deformity or genetic factors, in AIS patients as compared to healthy adolescents. Furthermore, their relevance in furthering understanding of the etio-pathogenesis of the disease is addressed.

## Materials and Methods

### Protocol

**Figure 1:**
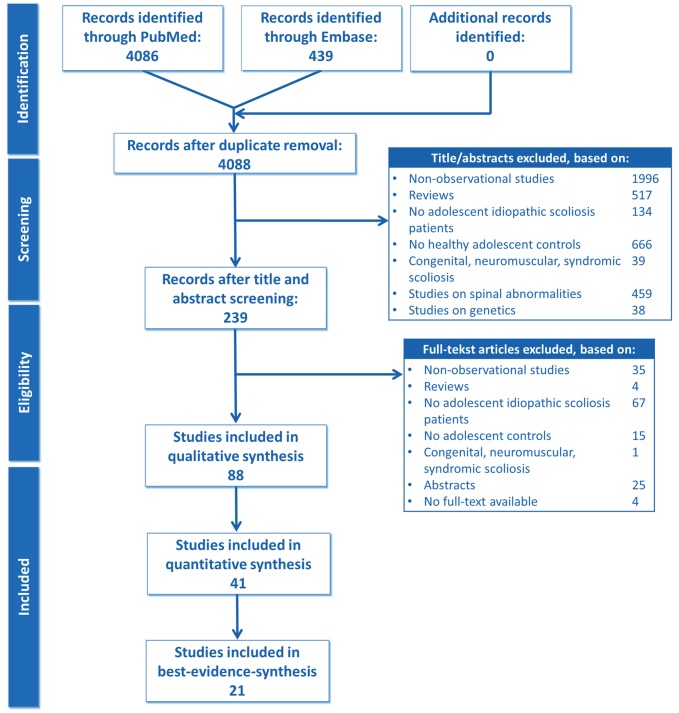
PRISMA flow diagram [13].

Following the PRISMA guidelines[13], we conducted a systematic review including the following subsequent steps: structured search, study selection, risk-of-bias assessment of individual studies and best-evidence-syntheses for relating risk-of-bias to consistency of effect sizes (figure 1). We did not register our review protocol.

### Search Strategy and Study Selection

Our search strategy was designed to ensure as wide a range of studies on AIS and associated abnormalities as possible. The search was conducted in PubMed and Embase digital databases in October 2013. We searched titles and abstracts using synonyms of idiopathic and scoliosis combined with Boolean operators (search syntax: “(idiopathic OR idiopathically) AND (scoliosis OR scoliotic OR scolioses OR spinal curvature)”. Included were English, Dutch and German full-text articles and there were no publication date or status restrictions. Duplicates were removed. Abstracts were checked for relevance by two independent investigators (TS and AV) using in- and exclusion criteria that were specified in advance. The investigators reviewed full-text articles when inclusion in the review was unclear from title and abstract. Uncertainties were discussed among the investigators. All original studies that reported a potential abnormality, other than the spinal deformity or genetics, in AIS patients compared to healthy adolescents were considered for inclusion. Studies on AIS patients who underwent treatment (bracing or surgery) or with congenital, neuromuscular or syndromes associated with scoliosis were excluded. Pathologies causing neuromuscular and syndromic scoliosis were predefined; neuromuscular scoliosis: cerebral palsy, syringomyelia, Chiari type-I malformation traumatic paralysis, spinal muscle atrophy, Friedrich’s ataxia, myelodysplasia, spina bifida, poliomyelitis or Duchenne’s disease[14]; Syndromic scoliosis: Marfan, Turner, Rett, Prader-Willi, Angelmann, Eagle-Barrett, β-thallasemia and 22q11.2 deletion syndrome, neurofibromatosis and osteogenesis imperfecta because development of scoliosis is common in these syndromes. Reviews and case series were also excluded. Reference lists from recent reviews, related articles as indicated by PubMed and reference sections of included articles were hand-searched for additional articles.

### Assessment of Risk-of-bias

For the remaining studies, risk-of-bias was assessed using a six-item risk-of-bias scoring system (minimal risk-of-bias score 0, maximal risk-of-bias score 6) that was specifically developed for our research question (Table 1).[15] For any disagreement between the observers, consensus was reached by discussion. All studies were ranked according to this score and articles with a total score ≥4 were included in the final analysis (low risk-of-bias: score 6; moderate risk-of-bias, score 4 or 5). We used a pre-defined cut-off value of ≤3 points (1/2 of the total score) in order to separate the high and moderate quality studies from studies with high risk-of-bias. Citations with a score ≤3 were excluded from our analysis.

**Table 1:** Risk-of-bias assessment was performed using a six-item scoring for description and validity of key information for the research question of this study and risk-of-bias.

Item	Scoring
***Selection:***	
1. Is the control grouprepresentative for normal adolescents?	1 = Community control; 0 = Hospital controls; 0 = No description of source
2. Was other pathology excludedthat possiblyinfluences the outcome?	1 = Yes; 0 = No or no description
***Comparability:***	
3. Were the same in- and exclusion criteria(except for the spinal deformity) used for AISand healthy adolescents?	1 = Yes; 0 = No or no description
***Exposure/outcome:***	
4. Were the observers blinded toAIS/healthy adolescent status?	1 = Yes; 0 = No or not documented
5. Was the data collection performed in thesame standardized way for AIS cases andhealthy adolescents?	1 = Yes; 0 = No or not documented
6. Was the primary outcomeparameter forAIS cases and healthyadolescents available?	1 = Available for >90%of AIS and healthy adolescents; 0 = Available for <90%of AIS or healthy adolescents

### Best-evidence-synthesis

**Figure 2:**
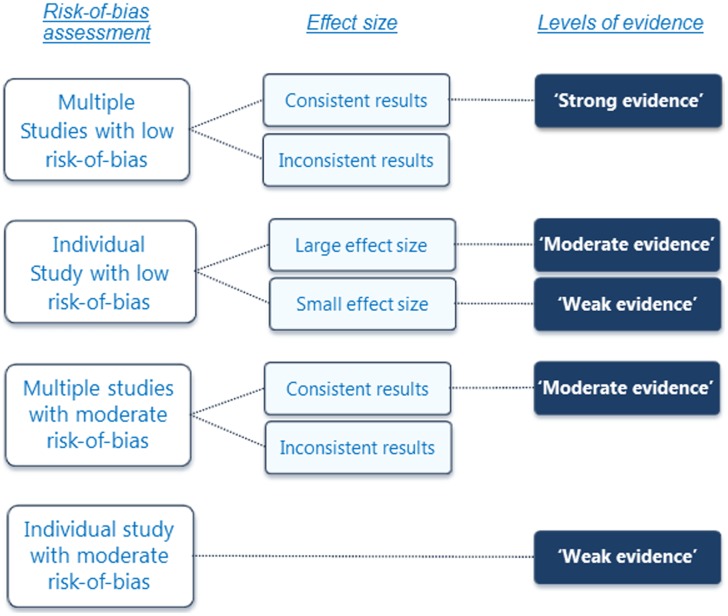
A best-evidence-synthesis was performed for each abnormality described in studies with satisfactory risk-of-bias. [16] Consistency was defined as ≥50% of the studies showed either a negative or positive effect of the primary outcome between AIS cases and healthy adolescents.

Due to great heterogeneity in abnormalities and outcomes of the different studies, we conducted a best-evidence-syntheses for each individual abnormality instead of a meta-analysis to quantify the relationship between each abnormality and AIS. This approach is the gold standard methodology for conducting qualitative and quantitative analyses of very heterogenic observational studies.[16] The synthesis was based on a combination of number of studies, risk-of-bias, consistency and size of the effect (figure 2). Source data (design, number of AIS subjects, number of control subjects, primary studied abnormalities, secondary differences between AIS and control cohort, mean outcome of AIS cases (µ_AIS_), mean outcome of healthy adolescents (µ_controls_), standard deviations (σ) or errors (se) and significance level were reviewed and extracted into tables by one investigator and checked by the other investigator. Effect size was calculated using Cohen’s *d* (Cohen’s *d* = (µ_AIS_–µ_controls_)/σ or (µ_AIS_–µ_controls_)/(se×√n). A qualitative approach to data synthesis was performed to assess the level of evidence for the association of each abnormality and AIS: Consistent findings in multiple papers with low risk-of-bias were rated as strong evidence; an individual study with low risk-of-bias and large effect size (Cohen’s *d*>0.8) or consistent findings in multiple studies with moderate risk-of-bias as moderate evidence; an individual study with low risk-of-bias and small effect size (Cohen’s *d*≤0.8) or with moderate risk-of-bias as weak evidence. If we were not able to calculate effect size based on poorly presented results, or conflicting findings were observed, the level-of-evidence was rated as insufficient. The literature was considered as consistent if ≥50% of the studies on different data sets showed either a negative or positive effect of the abnormality between AIS cases and healthy adolescents. For the best-evidence-synthesis, on one hand, research groups that reported on different abnormalities in multiple publications and investigated those in the same AIS dataset were considered as multiple studies. Because for most abnormalities the relation with other abnormalities is not very obvious (e.g. different vestibular organ morphometry, conus level and osteopenia), the risk of underreporting was avoided by this method. On the other hand, because of high-risk of overlap and to avoid duplicate (multiple) publication bias, multiple publications on the same abnormality studied by the same research group were counted as one study. If it was not clear whether the same dataset was used for different publications, for each abnormality only the publication with lowest risk-of-bias and largest sample size of each study group was included in the assessment of consistency of the literature. In addition, secondary outcomes were not included in the analysis to avoid duplicate (multiple) publication bias too. Risk of bias was addressed in this way, because we intended to give as complete an overview as possible of all abnormalities that have a consistent pattern of occurrence with AIS, based on the best available evidence.

## Results

### Search, Study Selection and Quality Assessment

The PRISMA flowchart is shown in figure 1.[13] The literature search yielded a total of 4525 publications. 441 duplicate entries were identified and removed. Most initial citations (n = 1996) were excluded because they did not offer any evaluation of abnormalities in untreated AIS patients, but described idiopathic scoliosis treatment outcomes. After title and abstract and full-text review, quality was assessed of eighty-eight publications. Ten (1.9%) discrepancies were found when the 528 (88 citations×six-items) scorings for risk-of-bias were compared between the independent observers (Cohen’s kappa = 0.962). These were resolved by discussion. We found substantial variation in risk-of-bias of reporting among the eighty-eight studies. A further forty-seven citations were discarded because they carried high risk-of-bias. Many of them did not contain description of the selection of the AIS cohort, as well as the healthy adolescent cohort.

Hence, forty-one citations were included in the quantitative synthesis. Another major limitation of the studies was found, which was not reflected in the risk-of-bias scores of the articles. Because descriptive outcome data were not reported in a sufficient manner in the full-text articles, the effect size and direction of effect could not be calculated for twenty articles. The level of evidence of these studies on potential abnormalities in AIS was ranked as ‘insufficient’. Ultimately, twenty-one publications that provided data on potential abnormalities in AIS were included in the best-evidence-synthesis.

### Study Demographics

The remaining twenty-one peer-reviewed publications all failed to report data from follow-up and blind assessments. All were cross-sectional observational studies and no longitudinal cohort or case-control studies were found (Table 2–4). Studies were conducted in a variety of sample sizes and subjects were enrolled in different continents. A total of 14451 AIS patients were compared with 94030 healthy adolescents. For individual studies, the number of included AIS patients and healthy adolescents varied between 10 and 11575 and between 10 and 92132, respectively. Fifteen (71%) studies recruited subjects in Asia, three (14%) in Europe and three (14%) in North-America. More specific, eleven (52%) studies were conducted by a single research group of the Chinese University of Hong Kong and four (19%) in other parts of Asia.

**Table 2:** Overview of articles identified in this study on neuromuscular concomitant abnormalities, sorted per category and ranked by risk-of-bias.

Authors	Study design	Total score	AISn	Con-trols n	Primary outcome parameter	Significant differences in secondary parameters	*P*	Cohen’s *d*
Shi et al.[17]	CS	6	20	20	Increaseddistance betweenvestibular canals	Angles between vestibular canals	0.03	−0.7
Guo et al.[18]	CS	5	57	105	Asymmetry ofsomatosensoryevoked potentials	Decreased arm muscle strength	0.03	−0.26
Kuo et al.[19]	CS	5	22	22	Improved gaitcontrol	-	NS	−0.50
Wang et al.[20]	CS	5	50	40	Thinner cortexright cerebrum	-	0.02	−1.13
Shi et al.[21]	CS	5	50	40	Decreasedvolumes rightand left cerebellarregions	-	0.03	−0.68
McIntire et al.[22]	CS	4	14	26	Decreased trunkmuscle strength	-	<0.05	−0.71
Bruyneel et al.[23]	CS	4	10	15	Impaired gaitcontrol	-	<0.01	0.62
Yang et al.[24]	CS	4	20	20	Impaired gaitcontrol	-	NS	1.36
Sun et al.[25]	CS	4	240	120	Level of the end ofthe spinal cord	-	NS	0.06
Gruber et al.[26]	CS	4	36	10	Impaired gaitcontrol	-	0.03	1.66

Abbreviations: AIS = adolescent idiopathic scoliosis; CS = cross-sectional; NS = not significant X = impossible to calculate Cohen’s d.

**Table 3:** Overview of articles identified in this study on anthropometic concomitant abnormalities, sorted per category and ranked by risk-of-bias.

Authors	Study design	Total score	AISn	Con-trols n	Primary outcome parameter	Significant differences in secondary parameters	*P*	Cohen’s *d*
Shohat et al.[27]	CS	5	11575	92132	Body height	Lower Body weight,lower body-mass-index	<0.001	0.18
Cheung et al.[28]	CS	5	621	300	Corrected body height	Lower body weight, lowerbone mass, longer armspan, longer leg length,lower bone mineraldensity, higher bonealkaline phosphatase	0.002	0.57
Barrios et al.[29]	CS	5	52	92	Body weight	Lower body-mass-index,higher Ponderal index,lower bony weight,	<0.05	−0.41
Normelli et al.[30]	CS	4	48	28	Breast asymmetry	-	<0.05	0.99

Abbreviations: AIS = adolescent idiopathic scoliosis; CS = cross-sectional; X = impossible to calculate Cohen’s d.

**Table 4:** Overview of articles identified in this study on metabolic concomitant abnormalities, sorted per category and ranked by risk-of-bias.

Authors	Study design	Total score	AISn	Con-trols n	Primary outcome parameter	Significant differences insecondary parameters	*P*	Cohen’s *d*
Lee et al.[31]	CS	5	619	300	Decreased bone mineral density	Lower body weight,increased corrected body height,lower body-mass-index,increased arm span,increased leg length,bone mineral content	<0.001	−0.94
Park et al.[35]	CS	4	19	16	Decreased bone mineral density	Decreased ability ofmesenchymal stem cellsfor osteogenicdifferentiation	0.037	−0.71
Lam et al.[36]	CS	4	635	269	Impaired bone quality	Lower body weight,increased corrected body height,increased arm span,lower body-mass-index,lower bone mineral density	<0.001	−0.42
Liu et al.[37]	CS	4	95	46	Lower serum leptin level	Lower body-mass-index,longer arm span,higher level of soluble leptin receptor,lower free-leptin-index	NS	−0.24

Abbreviations: AIS = adolescent idiopathic scoliosis; CS = cross-sectional; NS = not significant.

### Potential Abnormalities

In summary, fourteen potential concomitant abnormalities were studied as the primary outcome in twenty-one publications: Ten articles primarily focused on seven different neuromuscular abnormalities, four on four different anthropometric abnormalities and seven on three different metabolic abnormalities.[17]–[37] Two potential abnormalities were studied in multiple publications, whereas twelve were studied in one publication. The size of the effect varied between −1.13 and 1.36. Standardized risk-of-bias assessment showed that one study had the maximal risk-of-bias score of 6, and nine and eleven studies a score of 5 and 4, respectively. The common methodological limitation in the last studies with high risk-of-bias was absence or no documentation of blinding of the observers; one (5%) study described blinding of the observers for AIS/control status.[17] Three major groups of abnormalities emerged in the identified articles: neuromuscular, anthropometric and metabolic. In table 2, 3 and 4 all articles are shown, sorted per group and ranked by risk-of-bias score.

### Best-evidence-syntheses

**Figure 3:**
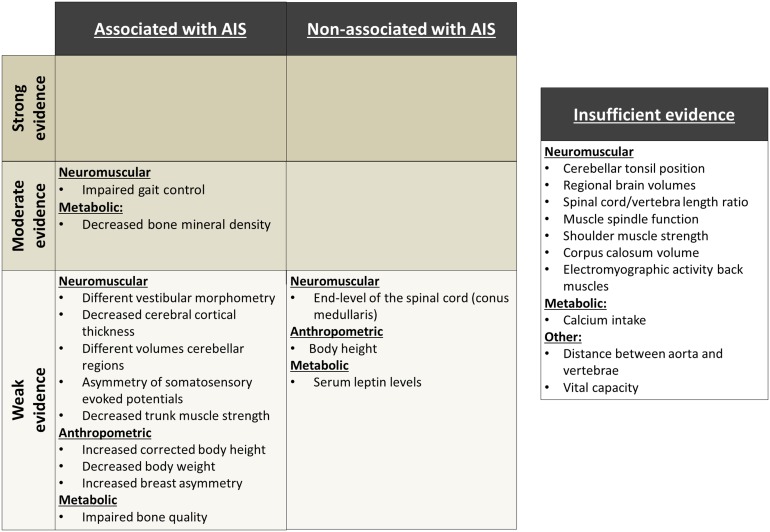
Level of evidence is shown for all associated and non-associated abnormalities that were identified in this systematic review. Level of evidence was determined using a best-evidence-synthesis. AIS = adolescent idiopathic scoliosis.

With our best-evidence-syntheses we were unable to find both strong evidence and a consistent pattern of occurrence for AIS for any of these abnormalities. Two out of fourteen potential abnormalities (gait control and bone mineral density) were described in multiple studies with moderate risk-of-bias. Despite the heterogeneity of outcome parameters in the different studies, direction of evidence and effect size were consistent and therefore, the evidence was moderate for association of these abnormalities with AIS. For nine other (neuromuscular, anthropometric or metabolic) abnormalities a consistent pattern of occurrence with AIS was found, but the evidence was weak. Moreover, three potential abnormalities were not significantly concomitant with AIS. Therefore, a total of eleven abnormalities were classified as associated with AIS with weak to moderate evidence (table 5; figure 3). In the following paragraphs, these abnormalities will be addressed.

**Table 5:** All identified abnormalities are presented.

Studied abnormality	Total numberof studies	Highestscore	Consistencyresults	Level ofevidence*	Associated with AIS?	Mean effect size
**Neuromuscular**						
Impaired gait control	4 (4†)	5	Yes	Moderate	Yes	1.00
Increased distance between vestibular canals	1	6	n/a	Weak	Yes	−0.70
Thinner cortex right cerebrum	1	5	n/a	Weak	Yes	−1.13
Decreased volumes cerebellar regions	1	5	n/a	Weak	Yes	−0.68
Asymmetry of somatosensory evoked potentials	1	5	n/a	Weak	Yes	−0.26
Level of the end of the spinal cord	1	4	n/a	Weak	No	0.06
Decreased trunk muscle strength	1	4	n/a	Weak	Yes	−0.71
**Anthropometric**						
Increased body height	1	5	n/a	Weak	No	0.18
Increased corrected body height	1	5	n/a	Weak	Yes	0.57
Decreased body weight	1	5	n/a	Weak	Yes	−0.41
Increased breast asymmetry	1	4	n/a	Weak	Yes	0.99
**Metabolic**						
Decreased bone mineral density	5(2†)	5	yes	Moderate	Yes	−0.83
Lower serum leptin level	1	4	n/a	Weak	No	−0.24
Impaired bone quality	1	4	n/a	Weak	Yes	−0.42

Abbreviations: AIS = adolescent idiopathic scoliosis; n/a = not applicable.

*Level of evidence was determined using a best-evidence synthesis.

† Number of studies from multiple research groups.

### Neuromuscular Abnormalities

Based on a large mean effect size, abnormal gait control and cortical thinning of the right cerebrum were the abnormalities most concomitant with AIS. Kuo et al., Bruyneel et al., Yang et al. and Gruber et al. compared dynamic balance control of AIS patients with normal adolescents using movable balance platforms or force plates.[19], [23], [24], [26] These four studies had moderate risk-of-bias. Overall, consistent results, a large mean effect size (1.00) and moderate evidence for an association of impaired gait control and AIS were found. In addition, the best available evidence was weak for abnormal morphometry of the vestibular organs, thinner cortex of the right cerebrum, decreased volumes of different cerebellar regions, asymmetry of somatosensory evoked potentials and decreased trunk muscle strength in AIS patients.[17], [18], [20]–[22] It was weak, because these abnormalities were each studied in one study. However, while the effect size of a thinner cortex of the right cerebrum between AIS patient and controls was large, it was small for the other abnormalities. In addition, weak evidence was found in a study of Sun et al. that the level of the lower end of the spinal cord (normally between T12 and L3) is not different between AIS cases and healthy adolescents.[25].

### Anthropometric Abnormalities

Shohat et al. showed in military records of 11575 17-year old AIS patients compared to 92132 non-scoliotic 17-year olds, the largest sample size that was found in this review, mean body heights of 163 and 162 cm, respectively.[27] The effect size, however, was very limited (Cohen’s *d*<0.2). From our systematic analysis, it was concluded that increased body height is not associated with AIS. Nonetheless, using Bjure’s formula, Cheung et al. showed increased body height in 13 to 15 year old AIS patients compared to healthy 13 to 15 year olds, when trunk height was corrected for the loss due to the coronal spinal deformity.[28], [38] In addition to increased corrected body height, Barrios et al. showed lower mean body weight in fifty-two AIS patients compared to ninety-two age-matched controls.[29] Further, Normelli observed significantly more left-right breast asymmetry in AIS patients compared to controls.[30] Because the last three abnormalities were studied in a single study, they were considered as associated with AIS, but the quality of the evidence was weak for all three. The relation between breast asymmetry and significantly concomitant with AIS was significant (mean effect size = 0.99), while the relation of AIS with increased corrected body height and decreased body weight was smaller (mean effect sizes 0.57 and −0.47, respectively).

### Metabolic Abnormalities

This review included seven different publications on four metabolic abnormalities. Assessment of the quality of the data suggests that evidence for decreased bone mineral density in AIS patients was moderate, for serum leptin levels and impaired bone quality weak, and for calcium intake insufficient. Four of five articles on bone mineral density were performed by the same group from Hong Kong.[31]–[35] Participant of these studies were included at the same scoliosis clinic or local secondary schools and inclusion periods were not documented. Due to high risk of overlapping datasets, three of their studies were not included in the best-evidence synthesis. The effect sizes of Lee et al. from Hong Kong (the study with lowest risk-of-bias and largest sample size) and Park et al. from Korea were used for evaluation of consistency of the identified literature on bone mineral density in AIS cases.[31], [35] Mean effect size of these two studies was large (−0.83). Therefore, decreased bone mineral density can be considered as significantly associated with AIS. In addition, impaired bone quality was also associated with AIS with a small effect size in a study of Lam et al., while serum leptin levels were not significantly associated with AIS in a study of Liu et al. [36], [37].

## Discussion

For better understanding of the etiology of AIS, this systematic review was designed to give as complete an overview as possible of extraspinal abnormalities that have been implicated to be concomitant with AIS. The outcome of our analysis concludes that, based on the available literature, strong evidence is lacking for a consistent pattern of occurrence of AIS and any abnormality. In addition, the abnormalities were never studied in AIS cases before the onset of the deformity. Weak to moderate evidence, however, was found for eleven abnormalities in AIS patients after the onset of the deformity. Based on a large mean effect size, four of these (impaired gait control, cortical thinning of the right cerebrum, increased breast asymmetry and decreased bone mineral density) can be considered as significantly concomitant with AIS.

After a critical review of the existing literature, a definite answer to the question that was raised in the title of this article cannot be provided. All publications on concomitant abnormalities had a cross-sectional design and the investigations were performed on AIS patients after the onset of the spinal deformity. Chronic deformation of the spine and asymmetry of the trunk can interact with many physiological systems outside the vertebral column.[2], [39] For most of the reported anomalies it remains unclear whether they occur during the development of scoliosis simultaneously with the spinal curvature and to what extent they also occur in scoliosis with a known cause, such as congenital and neuromuscular scoliosis.[40] For multiple abnormalities, including impaired gait control and decreased bone mineral density, significant correlations with Cobb’s angle or other severity measures has been documented, suggesting that they occur as a result rather than the cause of the deformity.[28], [31], [41] By studying populations with severe AIS curves, potential causal factors which disappeared after the spine started to grow crooked may have been missed. Thus, from an etiological perspective, the appropriateness of the cross-sectional study design of all studies that were identified is very limited. As an alternative, several researchers investigated the cause-and-effect relation of different abnormalities in animal models. For example, Machida et al. studied the onset of scoliosis in pinealectomized chickens and Lambert et al. in frogs after unilateral vestibular organ removal.[42], [43] It can be questioned, however, whether findings in animal models with iatrogenic scoliosis can be translated to humans, because natural development of idiopathic scoliosis cannot be found in any other vertebrate than man, and no invasive procedures are normally required to cause it.[44]–[46] Therefore, the identified data of cross-sectional studies as well as data from animal models do not clearly support the hypotheses that the described abnormalities play a role in the etio-pathogenesis of idiopathic scoliosis.

Since all neuromuscular disorders that act on the growing body by impaired gait control inevitably lead to the development of scoliosis, it has been inferred that idiopathic scoliosis is the result of a ‘forme fruste’ of neuromuscular disease. This has started in the 1940s with the suggestion that a latent form of poliomyelitis would play a role.[47], [48] Many patients with idiopathic scoliosis, however, function very well, often also athletically, up to the moment that they reach puberty and develop a curvature of the spine. After cessation of growth they very often do not manifest any other abnormality than their spinal deformity, and certainly no neurologic disability. In fact, it is increasingly recognized that instead of extrinsic neuromuscular factors the unique intrinsic spinal biomechanics of the upright human spine and genetic predisposing factors play a key role in multifactorial initiation of different pediatric spinal deformities, such as idiopathic scoliosis [9]–[12].

We searched for evidence describing any consistent abnormality, other than the deformity of the spine, in AIS patients compared to normal adolescents. Due to publication bias, it is likely that significant anomalies in AIS patients are more often reported in the literature than normal findings. In addition, most potential anomalies have been studied by an individual research group that was probably focused on one or more potential anomalies to test their own etiological hypothesis, providing high risk of confirmation bias. In this structured review we tried to minimize risk-of-bias by performing a best-evidence-synthesis. By this synthesis, we based our consideration of the directness of evidence on the appropriateness of the quality for our research question (validity), size of the effect and consistency of finding of studies with relatively lowest risk-of-bias.[16] It was observed, however, that for almost half of the identified articles (20 of 41) on this topic, descriptive statistics were poorly presented, effect size could not be calculated and that only statistical significance between AIS subjects and controls was documented. The reported statistical significance, however, is not a direct parameter for effect size, but rather a function of effect and sample size.[49] Because studies without complete descriptive data may have found significant differences with a completely irrelevant effect size, these studies were not included in the final analysis. In summary, systematic analysis of the best available data showed that several abnormalities that were initially described as associated with AIS in the literature, were classified as not associated with AIS, or as ‘insufficient evidence’ after the critical evaluation. Another limitation of the literature on potential abnormalities in AIS patients was that for nine associated abnormalities, only one study was found and most of these studies were performed by a single research group. It cannot be derived from our data whether multiple abnormalities were found in the same study population and have confounding relations, and whether demographic factors played a role.

The disorder that we call ‘idiopathic’ scoliosis is far from homogeneous, and spinal rotatory decompensation into deformity seems a rather aspecific response to a multitude of factors. From an epidemiological perspective, Rothman et al. described a model to address problems of multifactorial causation, the sufficient-component cause model. In short, “*the causal mechanism for any effect must consist of a constellation of components that act in concert*”.[50] In AIS, the causal mechanisms (also known as “sufficient cause”) as well as the necessary and complementary components for initiation of these mechanisms are not well understood. The diversity in components of causal pathways in AIS has complicated investigations into the multifactorial origin of idiopathic scoliosis in the past and probably will continue to do so in the future. Because of its multifactorial origin, multivariate testing is needed in studies on components of sufficient causes of AIS. Nevertheless, most studies that were identified in this review focused on a unique causal factor of AIS and used univariate analyses for this reason. Therefore, their findings may be statistical artifacts. So, to make causal inferences we should take account of the constellation of multiple components in the causal pathways of AIS, by taking account of the strength of the effect and timing of concomitant abnormalities.

## Conclusion

Several subtle abnormalities exist in AIS patients. However, the relevance of these associated disorders for understanding the etio-pathogenesis of AIS is very limited. For now, it seems that the development of scoliosis is the growing spine’s pre-conditioned response to a multitude of unknown offenses to disturb the delicate human rotational spino-pelvic balance during growth.[7], [8], [44] Understanding the relevance of the phenotypical heterogeneity of AIS patients is of great value for scientists that focus on the etio-pathogenesis of AIS.

## Supporting Information

Checklist S1 **PRISMA 2009 Checklist for systematic reviews and meta-analyses.**
(DOC)
